# Brno urban dataset: Winter extension

**DOI:** 10.1016/j.dib.2021.107667

**Published:** 2021-12-03

**Authors:** Adam Ligocki, Ales Jelinek, Ludek Zalud

**Affiliations:** Brno University of Technology, Technicka 12, Brno 612 00, Czechia

**Keywords:** Multimodal dataset, Navigation data, RGB camera, IR camera, 3D LiDAR, RTK GNSS, IMU, Neural networks

## Abstract

This paper presents our latest extension of the Brno Urban Dataset (BUD), the Winter Extension (WE). The dataset contains data from commonly used sensors in the automotive industry, like four RGB and single IR cameras, three 3D LiDARs, differential RTK GNSS receiver with heading estimation, the IMU and FMCW radar. Data from all sensors are precisely timestamped for future offline interpretation and data fusion. The most significant gain of the dataset is the focus on the winter conditions in snow-covered environments. Only a few public datasets deal with these kinds of conditions. We recorded the dataset during February 2021 in Brno, Czechia, when fresh snow covers the entire city and the surrounding countryside. The dataset contains situations from the city center, suburbs, highways as well as the countryside. Overall, the new extension adds three hours of real-life traffic situations from the mid-size city to the existing 10 h of original records. Additionally, we provide the precalculated YOLO neural network object detection annotations for all five cameras for the entire old data and the new ones. The dataset is suitable for developing mapping and navigation algorithms as well as the collision and object detection pipelines. The entire dataset is available as open-source under the MIT license.

## Specifications Table

**Table:** 

Subject	Computer Science, Artificial Intelligence
Specific subject area	It is a dataset for mapping and autonomous agent development.
Type of data	RGB and IR images, point cloud scans, text data logs
How data were acquired	Real-life traffic recording, using the sensory framework mounted on the roof of the car. The framework employs four 1920 × 1200px@10fps RGB cameras, single thermal (IR) camera 640 × 512px@30fps, two Velodyne HDL-32E LiDARs, a single Livox Horizon one, BX982 RTK GNSS receiver with differential antennas, Xsens MTI-G-700 IMU, and the TI mmWave 1648 FMCW radar.
Data format	Raw sensor data are stored in three forms. The camera data are stored as an mp4, h265 video. The LiDAR stans are stored as .pcd scan files. There is a corresponding text file for both data types that describes when the data were captured. The rest of the data are stored as a CSV record that describes the timestamp and the measured values. Additionally, we split the dataset into several recording sessions. Each session takes place in a dedicated folder.
Parameters for data collection	Dataset is mainly focusing on real-life traffic in the winter conditions.
Description of data collection	We organize the experiment, by mounting the sensory framework on the car's roof and recorded data from ten sensors during the 90 km ride in the Brno, during winter in the late February of 2021. In total, we provide about 3 h of raw data logs from the city centre, highways, urban areas, and countryside.
Data source location	Czechia, Brno, 49.195N, 16.608E
Data accessibility	Data are available on: https://github.com/Robotics-BUT/Brno-Urban-Dataset
Related Research Articles	Brno Urban Dataset (Ligocki et al. 2020) https://ieeexplore.ieee.org/abstract/document/9197277 Fully Automated DCNN-Based Thermal Images Annotation Using Neural Network Pretrained on RGB Data (Ligocki et al. 2021) https://www.mdpi.com/1424-8220/21/4/1552

## Value of the Data

• The dataset contains state-of-the-art data from sensors commonly used in robotics or autonomous driving, like RGB and IR cameras, 3D LiDARs, diff. RTK GNSS, IMU, or the FMCW radar. All data are timestamped.
• Dataset focuses on winter conditions and includes sessions from the snow-covered city center, suburb areas, countries, and highways. It is an ideal data background for developing sensory fusion, obstacle detection, mapping, and localization algorithms.
• The dataset contains precalculated object detection annotations by YOLO neural network for all camera data. In total, the 13 h of detections on 4 RGB and 1 IR camera data (3.3mil images). It allows processing data in real-time, even on computers without high-performance GPUs.
• Data was already used during the work on scientific projects [10].

## 1 Experimental Design, Materials and Methods

### 1.1 Sensory equipment

The original setup contains four RGB cameras with 1920 × 1200 px resolution (Imaging Source DFK-33GX174 with 1/1.2 inch Sony IMX174 chip) and used at the 10 Hz sampling rate. Two cameras are directed forward, and each covers approximately 70° FoV (f=8 mm/F1.4 lens). Two lateral cameras cover the car's sides with 90° FoV (f=6 mm/F1.8 lens). All RGB cameras combined cover about 220° FoV around the car.

The RGB vision is enhanced with a single near-infra-red thermal camera FLIR Tau2 with 640 × 512 px resolution and 30 Hz frame rate. The camera senses radiation in a 7.5–13.5 µm spectrum that corresponds with a common objects' temperature in the range of -40 to 80°C. This type of sensor provides an advantage in bad light conditions, as it senses the infra-red radiations that each object emits on its own, without a need for an external light source. Therefore, it is especially useful for living creatures or heated car detection. The camera is equipped with a 9 mm lens, that provides 70° FoV.

Next, we employed two Velodyne HDL-32 LiDARs with 32 laser beams, which sense 2000 points per laser beam per scan with a rate of 10 Hz. In total sensor produces about 0.6 mil points per second. Both LiDARs are slightly tilted around the forward-pointing axis. First, it gives a better cover of areas near the car. Second, as the sensors sense in different plains, they create a grid-like pattern on objects visible by both sensors and better cover them with range measurements.

The fourth sensor is the Xsens-MTi-G-710 3D inertial measurement unit that provides the 3D linear acceleration, 3D angular velocity, 3D magnetic field measurement, temperature, and air pressure. Additionally, IMU also receives low-quality GNSS data. Last but not least important sensor in the original setup is the Trimble BX 982 RTK GNSS receiver with differential Trimble AG25 antennas enabling heading estimation.

**Fig. 1:**
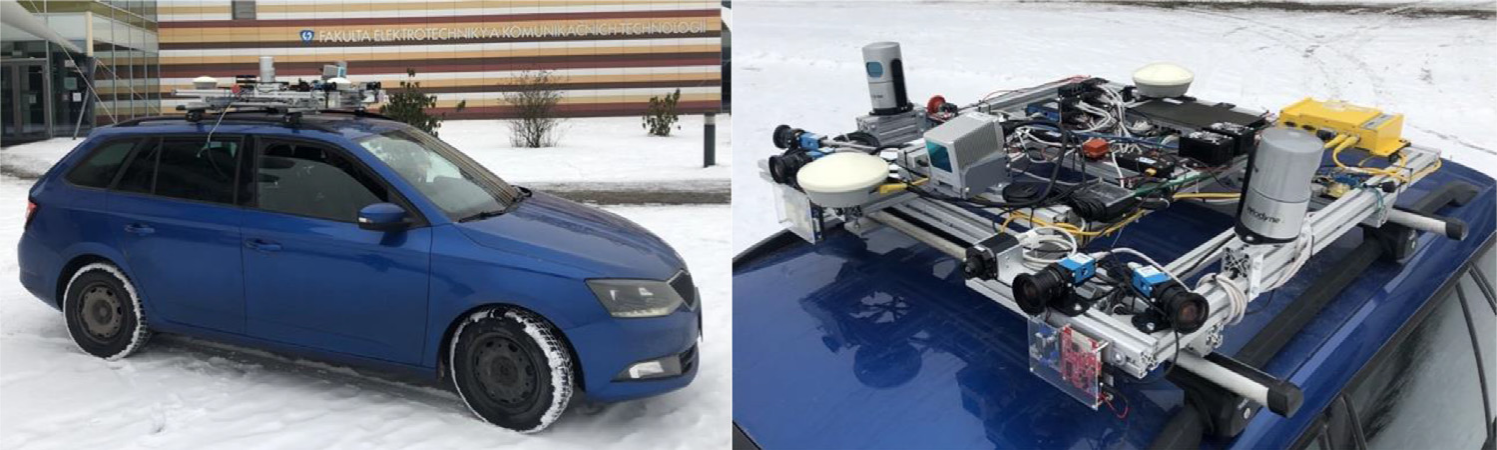
(left) The Atlas sensory framework installed on the roof of the testing car. Data were recorded in the early February of 2021; (right) Detail sensory framework overview. Four RGB cameras (blue), two side Velodyne scanners and single Livox sensor (all gray), GNSS RTK receiver (yellow) with pair of differential antennas (white), Xsens IMU (orange) in the center, and the FMCW radar (red) in the front of the framework.

**Fig. 2:**
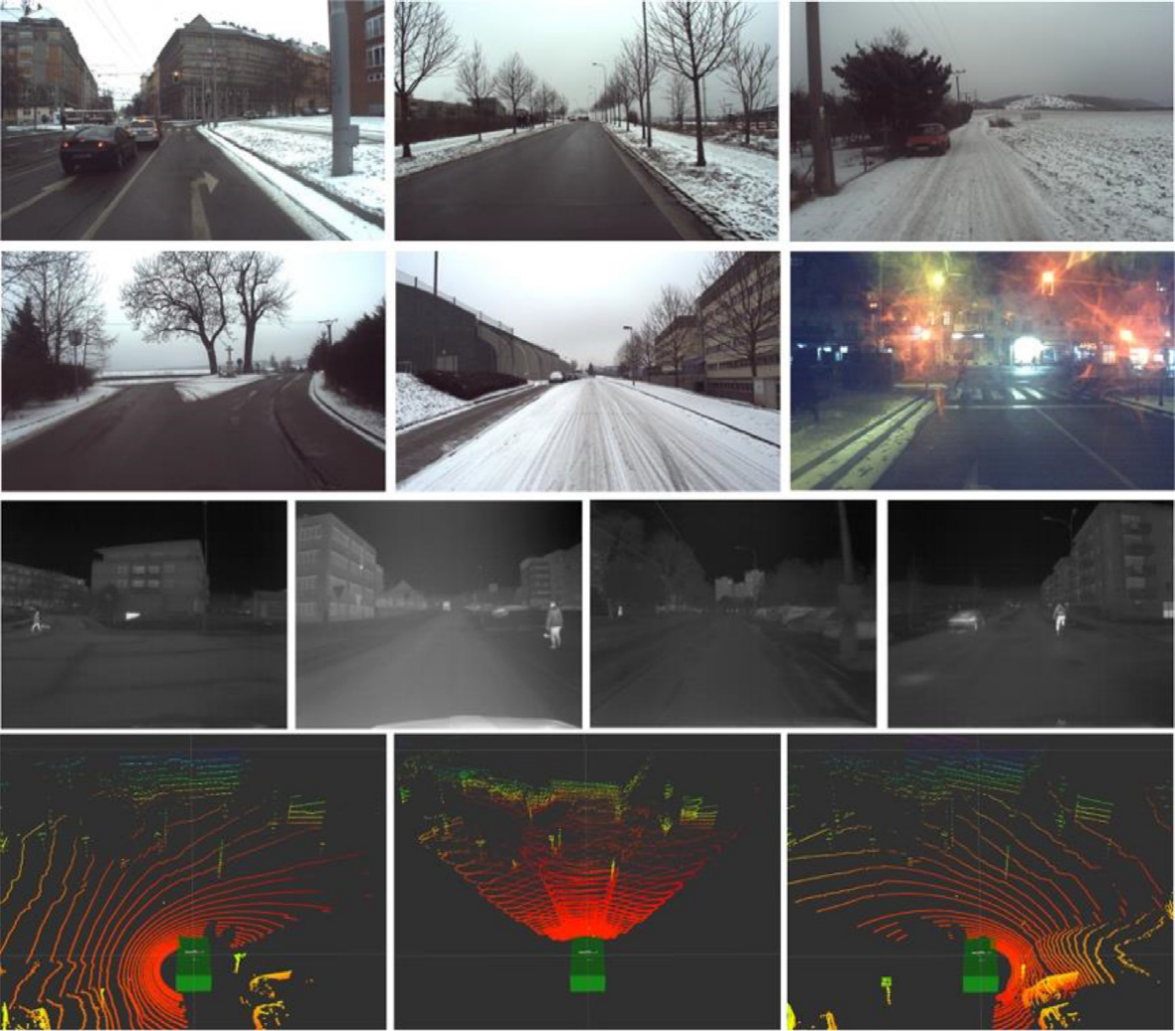
Sensors used overview: RGB cameras (top two rows), IR camera (third row), and LiDARs (bottom line - left Velodyne, forward-looking Livox, and right Velodyne scanners).

Fig. 1 shows the data acquisition platform and its installation on the roof of the car. Fig. 2 shows the sample data from the recorded sensors.

Newly, we mounted a single Livox Horizon LiDAR on the front of the sensory framework, covering approximately 80° horizontal and 25° vertical FoV in front of the car. The sensor generates about 240 000 points per second with 2 cm and 0.05° angular accuracy. According to the official documentation, the LiDAR senses objects up to 260 m away. In our case, the objects can be seen by the LiDAR with high certainty at a distance of 150 m.

In contrast to the Velodyne HDL-32e, the Livox Horizon does not scan the entire 360° surrounding with multiple laser beams, but it uses a single 905 nm laser that continuously covers the sensor's frontage with a non-repetitive, rose-like pattern. This setup greatly improved point cloud density in the most crucial region in front of the vehicle.

The Livox Horizon's data are stored in the *lidar_center/* subfolder of the recording in a similar format as the Velodyne LiDAR data. As the Horizon sensor scans the non-repetitive pattern and provides data as a continuous stream of points. We split this stream into 100 ms, time-ordered chunks, which are saved as .pcd scans in a common .zip file with the corresponding timestamp .txt file.

For radar sensing, we used the TI mmWave 1642 model. A solid-state Frequency-Modulated Continuous Wave (FMCW) radar uses an antenna array and frequency modulated bursts to detect objects in front of the device. Gao et al. [5] describes the measurement principle in detail. The great advantage of the radar sensor is that the sensor provides the positioning of the detected object but using the Doppler's effect also measures the radial velocity of the object with respect to the radar.

In our setup, the radar is configured as a 2D sensor with a scan rate of 30 Hz. It provides data of *x, y* coordinates of the detected object and its radial velocity with respect to the radar sensor. The maximal range of the detected object is 50 m, with 0.97 m resolution, and the maximal radial velocity is ±18.5 ms^−2^ with 0.58 ms^−2^ resolution. Also, the radar senses object independently in light conditions, and it works even during the rain or fog when the cameras are blinded.

Each scan is stored in the *radar_ti/* subfolder of the recording session folder in a .txt file. There is a timestamp of the scan on every line and a count of detected objects *N* continued by 4*N* numbers representing *x, y*, and *z* coordinates of the detected objects and their radial velocity. The z coordinate is always zero in our case, but we chose this format for future compatibility.

### 1.2 YOLO detections

We also included precalculated YOLOv5 [6] object detections for all RGB camera data and the IR detections by our IR-data-trained YOLO neural network [7] to the BUD: WE (Brno Urban Dataset: Winter Extension). These additions were done not only for the newly recorded data but also retroactively for both previously published sessions published by [1].

For generating the RGB object annotations, we used the pre-trained neural network on the COCO dataset [8], covering 80 different object classes. For automotive applications, the most important classes are persons (0), vehicles and bikes (1–7), traffic signs (9,11), and living creatures (14–23). For IR images annotation, we used the neural network trained according to the [7]. The annotated classes are pedestrians (0), bikes (1), vehicles (2), and dogs (16).

We made a dedicated .txt file with the camera's name in the */yolo* subfolder for every recording session, describing a single object per line. Each line contains information about the detected object's class, the object's bounding box size and location in the image, and the detection confidence. We recorded all detections with confidence greater than 0.4 and intersection over union with other detection greater than 0.2.

**Fig. 3:**
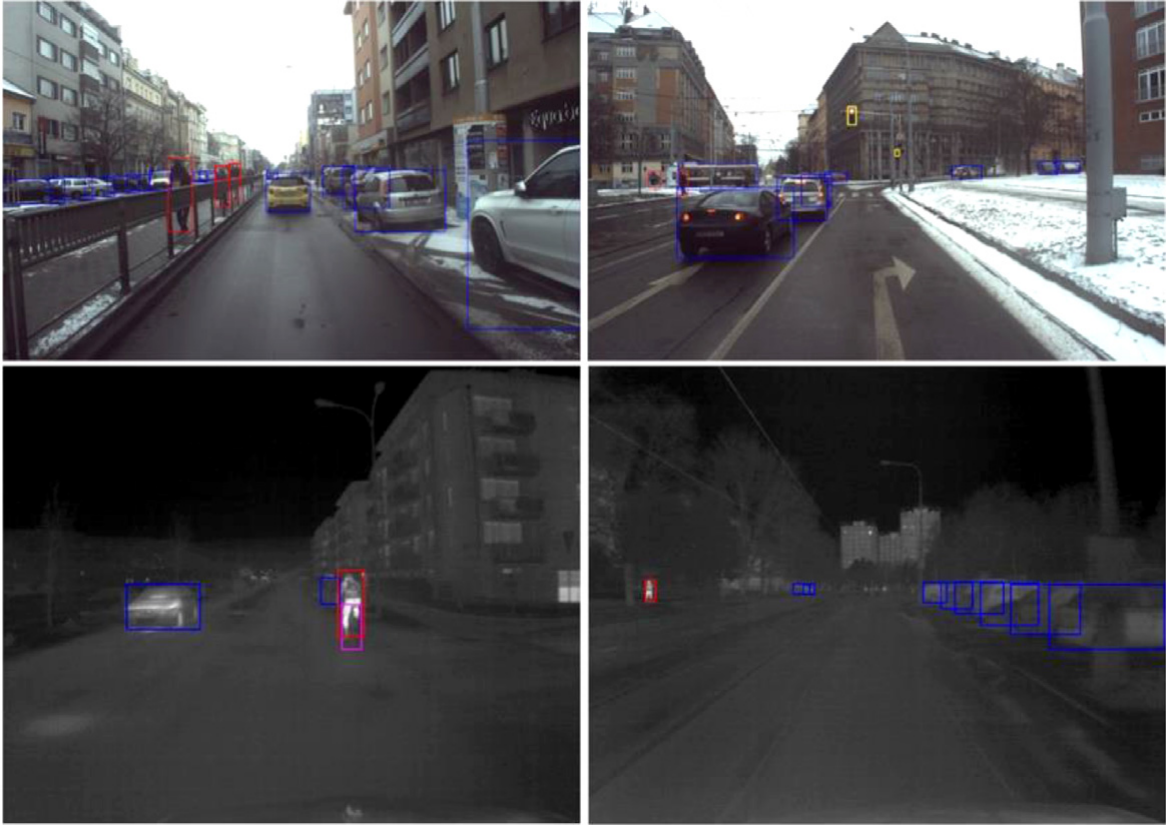
Example of neural network precalculated labels for RGB and IR camera data. In total, we provide annotations of approximately 52 h of RGB video (13 h for four cameras) at ten fps and about 13 h of annotated IR video. In total, it makes about 3.2.

**Fig. 4:**
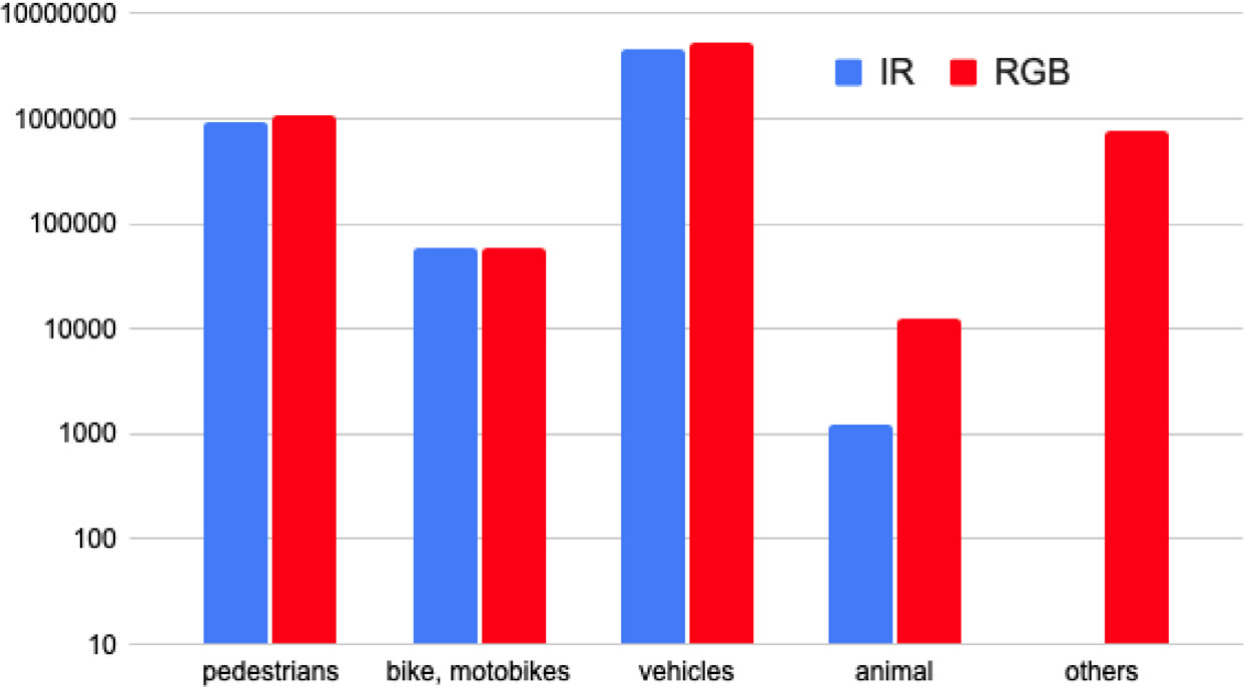
Overview of the most common annotated objects in the RGB and IR domain.

The example of neural network detection is shown in Fig. 3. Fig. 4 shows the distribution of the annotated classes in the image domain.

### 1.3 Time synchronization

To keep all sensors synchronized, we used the GNSS time frame for all devices that receive it. Specifically, these are the RTK GNSS receiver, the IMU, and both Velodyne LiDARs. The data capture computer is synchronized directly with the GNSS receiver by the NTP time server that runs on the receiver. To synchronize the RGB cameras, we created a dedicated microprocessor board that receives PPS signal and time data via serial bus from the GNSS receiver and handles precise camera triggering and timestamp logging. The Livox Horizon synchronization is handled by the PTPv2 time synchronization between the sensor and the data-gather computer. The only two sensors that we could not precisely timestamp are the thermal camera and the radar, as they do not provide an input or output triggering signal with the current firmware version.

### 1.4 Calibration

To estimate the calibration parameters of the sensors, we used several publicly available tools. For RGB cameras and IMU parameter estimation, we used the Kalibr tool [2] and [3] to estimate the transformation between the RGB cameras and LiDARs. For thermal camera position estimation, we follow the work [4]. The positions of GNSS antennas are estimated based on the hardware documentation and relation between the antenna's chassis mount and the actual position of the antenna inside the chassis.

### 1.5 Backend infrastructure

The data gathering system's core is a battery-powered desktop computer with AMD Ryzen Threadripper 1950x CPU, Nvidia 1080Ti graphic card, and NMVe SSD disk for high data bandwidth necessary for recording. The 800 Wh battery pack can supply the computer and the entire sensory framework for about two hours. The RGB and thermal cameras, LiDARs, GNSS receiver, and camera synchronization microcontroller board are connected via a D-Link DGS-1510-20 IP switch with a 10Gb/s line to the computer. The IMU is connected through USB, which simulates a virtual serial bus.

### 1.6 Recordings

In total, we extended our existing 10 h and 350 km dataset with four new recording sessions that cover approximately 3 h and 90 km of new real-traffic data in snowy winter conditions. Three sessions are recorded during daylight in cloudy weather. They cover city rides as well as high-speed roads, countryside, or dense urban localities and several loop-closing scenarios.

**Fig. 5:**
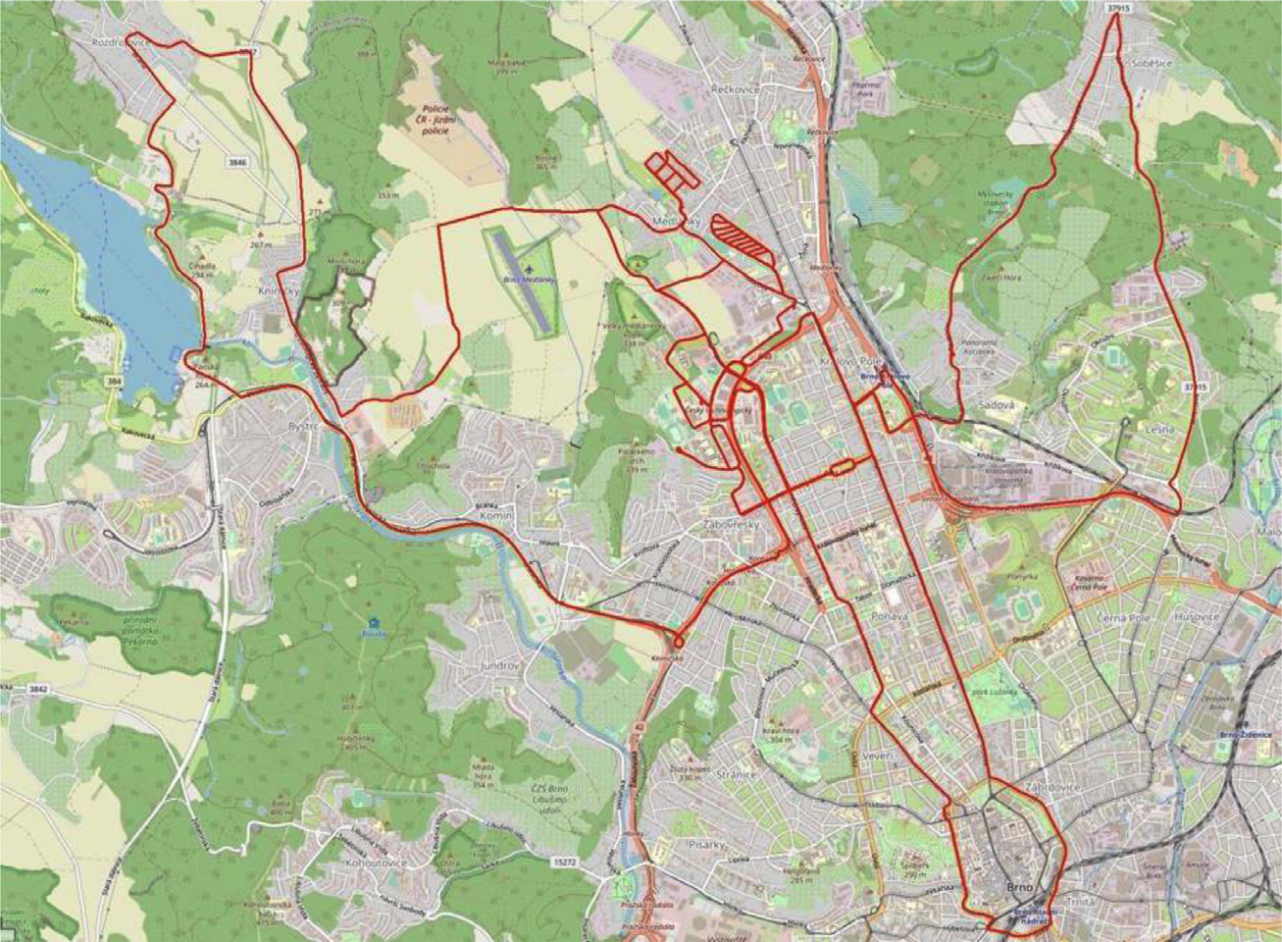
Visualized trajectories traversed during the BUD Winter Extension recording in Brno, Czech Republic.

The fourth session is recorded after the sunset in an early night. The freezing rain cached us during the last recording session that covered sensors and nearly completely blinded all camera sensors in several minutes. This session nicely represents harsh environmental conditions, which an automated vehicle can face in practice. The sensors' reliability is still an unsolved problem. For example, the snow and mentioned freezing rain can be handled with the heated cover of the sensor. On the other hand, the water that remains on the sensor's optics still affects the measurement. Moreover, in conditions of strong winter and high velocity of the vehicle, even the sensor's heating can fail. This type of corrupted data allows developers to work on algorithms that dynamically evaluate the sensors' reliability and adjust the decision-making pipeline. These four sessions are divided into 13 shorter parts. See Table 1 for details and Fig. 5 depicting traversed trajectories.

**Table 1:** Brno urban dataset: winder extension recording sessions overview.

Session	Part	Env.	Distance [km]	Duration [h:mm]	Daytime and Weather
1	1	sub	8,2	00:17	noon - cloudy
	2	city	13,5	00:30	

2	1	country	6,8	00:15	noon - cloudy
	2		21,3	00:31	

3	1	sub	1,4	00:03	afternoon - clody
	2		8,9	00:20	
	3		1,5	00:03	
	4		4,7	00:11	
	5		3,3	00:07	

4	1	sub	2,8	00:07	night - soft rain
	2	sub	2,4	00:06	
	3	country	3,7	00:07	
	4	sub	8,7	00:12	

Total	13	-	87,2	02:50	-

## 2 Data Description

Each recorded session part has a strict structure and format of all recorded data. For a detailed description, please see Table 2.

**Table 2:** Datafiles structure and description

Subfolder	File	File Description; Data Format
calibration\	camera_ir.yaml	Contains width and height of the image and intrinsic calibration parameters, and camera distortion parameters
	camera_left_front.yaml	
	camera_left_side.yaml	
	camera_right_front.yaml	
	camera_right_side.yaml	
	frames.yaml	Defines translation and rotation for each sensor with respect to the center of the frame (the IMU sensor)
camera_ir\	video.mp4	512 × 640px@30fps, h265 video
	timestamp.txt	Video frames timing and min and max temperature on the IR image [°C]; Line format: epoch_timestamp, frame_no, minimal_temp, maximal_temp
camera_left_front\	video.mp4	1920 × 1200px@10fps, h265 video
camera_left_side\		
camera_right_front\	timestamp.txt	Video frames timing and internal camera's timing; Line format: epoch_timestamp, frame_no, camera_timestamp
camera_right_side\		
gnss\	pose.txt	WGS84 position; Line format: epoch_timestamp, latitude, longitude, altitude, azimuth
	time.txt	Time form IMU's GNSS receivee; Line format: epoch_timestamp, year, month, day, hours, minutes, seconds, nanoseconds
imu\	d_quat.txt	Sensor's orientation difference from the last sample as a quaternion; Line format: epoch_timestamp, quat_x, quat_y, quat_z, quat_w
	gnss.txt	WGS84 position by IMU's gnss receiver; Line format: epoch_timestamp, latitude, longitude, altitude
	imu.txt	3D linear acceleration [m/s^2^], angular velocity [rad/s], and IMU's orientation as quaternion;
		Line format: epoch_timestamp, acc_x, acc_y, acc_z, ang_vel_x, ang_vel_y, ang_vel_z, quat_x, quat_y, quat_z, quat_w
	mag.txt	3D magnetic induction [G]; Line format: epoch_timestamp, mag_x, mag_y, mag_z
	pressure.txt	Atmospheric pressure [Pa]; Line format: epoch_timestamp, pressure
	temp.txt	Sensor's temperature [°C] Line format: epoch_timestamp, temerature
	time.txt	Time from GNSS receiver; Line format: epoch_timestamp, year, month, day, hours, minutes, seconds, nanoseconds
lidar_center\	scans.zip	Zip file that contains all .pcd scan files. Each .pcd file contains a 100 ms data chunk
	timestamp.txt	Timing for LiDAR scans; Line format: epoch_timestamp, scan_number
lidar_left\	scans.zip	Zip file that contains all .pcd scan files. Each .pcd file contains data from a single full 360 deg scan by the LiDAR sensor
lidar_right\	timestamp.txt	Timing for LiDAR scans; Line format: epoch_timestamp, scan_number, inner sensor's timestamp
radar_ti\	scans.txt	Objects detected by the FMCW radar, position [m] and radial velocity [m/s] for each object;
		Line format: epoch_timestamp, no_of_detected_object, [x_0, y_0, z_0, radial_vel_0, x_1, y_1, ..., radial_vel_n]
yolo\	camera_left_front.txt	Objects detected in the camera data; Line format: frame_number, bb_center_x, bb_center_y, bb_width, bb_height, detection_confidence, class

### 2.1 Data structure

Each recording session appears in a separate folder, where each sensor stores data in the dedicated folder. RGB data are stored as a .mp4 videos with lossless h265 encoding and a corresponding .txt file that contains timestamps for each camera frame. We chose this video setup to keep the high quality of distributed image data, and at the same time to keep the decent size of the dataset. The lossless preset of the h265 encoding means that the encoding process bypasses the DCT (discrete cosine transformation) and the quantization, but the predictions are still used. The LiDAR point clouds are stored in a .zip file as a set of .pcd files for each 360° scan. Again, each scan has the corresponding timestamps in its accompanying .txt file. For Livox LiDAR, each .pcd file contains the set of points measured during the 100 ms time chunk. Finally, the IMU, radar, and GNSS receiver store data in text format in separate files, where each file corresponds to a specific data type (acceleration, angular velocity, global pose, etc.).

In the case of calibration, there is a file dedicated to each camera that contains its intrinsic calibration parameters and distortion coefficients. Another text file contains sensor layout information as a translation and rotation with respect to the IMU.

The main inspiration for chosen data format was the Oxford RobotCar Dataset [9].

## Ethics Statement

The authors declare that the manuscript meets all the rules and conditions described in the “Ethics in publishing” section (https://www.elsevier.com/journals/data-in-brief/2352-3409/guide-for-authors). During the dataset gathering, no experiments on humans nor animals were involved. All the data was collected in accordance with the law of Czechia.

## CRediT authorship contribution statement

**Adam Ligocki:** Visualization, Conceptualization, Data curation. **Ales Jelinek:** Visualization, Conceptualization, Data curation. **Ludek Zalud:** Supervision.

## Declaration of Competing Interest

The authors declare that they have no known competing financial interests or personal relationships which have or could be perceived to have influenced the work reported in this article.
